# Towards Real-Time Monitoring of Thermal Peaks in Systems-on-Chip (SoC)

**DOI:** 10.3390/s22155904

**Published:** 2022-08-07

**Authors:** Aziz Oukaira, Ahmad Hassan, Mohamed Ali, Yvon Savaria, Ahmed Lakhssassi

**Affiliations:** 1 Electrical Engineering Department, Polytechnique Montreal, Montreal, QC H3T 1J4, Canada; 2 Microelectronics Department, Electronics Research Institute, Cairo 12622, Egypt; 3 Department of Engineering Computer Science, University of Québec in Outaouais, Gatineau, QC J8X 3X7, Canada

**Keywords:** Field-Programmable Gate Array (FPGA), Integrated Circuits (ICs), System on Chip (SoC), Gradient Direction Sensor (GDS), thermal camera, thermal monitoring, thermal peak

## Abstract

This paper presents a method to monitor the thermal peaks that are major concerns when designing Integrated Circuits (ICs) in various advanced technologies. The method aims at detecting the thermal peak in Systems on Chip (SoC) using arrays of oscillators distributed over the area of the chip. Measured frequencies are mapped to local temperatures that are used to produce a chip thermal mapping. Then, an indication of the local temperature of a single heat source is obtained in real-time using the Gradient Direction Sensor (GDS) technique. The proposed technique does not require external sensors, and it provides a real-time monitoring of thermal peaks. This work is performed with Field-Programmable Gate Array (FPGA), which acts as a System-on-Chip, and the detected heat source is validated with a thermal camera. A maximum error of 0.3 °C is reported between thermal camera and FPGA measurements.

## 1. Introduction

The evolution of integrated circuits (ICs) has led to the design of increasingly dense circuits to allow implementing much more complex systems in a smaller silicon area [1]. This allows for reducing manufacturing costs and boosting systems’ performance. However, chips with high integration density dissipate high power, which consequently induces overheating problems that can cause disastrous thermal peaks. Thus, an appropriate management of thermal dynamics including thermal monitoring is required to avoid performance degradation and lifetime reduction of ICs [2,3,4]. On-chip thermal behavior can be obtained by adopting an on-chip distributed oscillator network [5,6], where the frequency variations generated by the integrated oscillators indicate the thermal changes in the chip. Then, using the Gradient Direction Sensor (GDS) technique the authors could monitor the temperature source of the thermal sensors on the surface of the chip [7]. This source implies isotherms around it. The GDS method is a practical solution to locate the thermal peak in the simplest case of a single heat source [8,9], and the functional range of this GDS method is between −55 °C and 125 °C because, as the supply voltage decreases, the temperature dependence of the propagation delay can change from negative to positive for supply voltage levels between 0.7 V and 1.2 V [10,11]. However, the measured temperatures are not processed in real-time, which has an impact on the accuracy of the readings. In order to improve this accuracy, we propose in this work to collect and process the temperatures in real-time based on FPGAs. Therefore, FPGAs offer the possibility to perform real-time simulation and implementation with a time step of tens of nanoseconds in addition to the real-time reconfiguration that dynamically modifies the memory content of an FPGA [12].

In this paper, we propose a method that aims to detect thermal spikes in SoC using oscillator arrays distributed on the chip surface. An indication of the local temperature of a single heat source is obtained using the GDS technique. The measured frequencies are mapped to local temperatures which are used to produce a thermal map of the chip. The proposed technique does not require external sensors.

This work is validated with a Field-Programmable Gate Array (FPGA) implementation and temperature measurements performed with a thermal camera. A maximum error of 0.3 °C is reported between thermal camera and FPGA measurements. This is significant as the majority of designers of ICs and system-on-chips (SoCs) do not have effective means of predicting an internal thermal cartography in real-time.

This paper is organized as follows: Section 2 describes the theory of operation of the on-chip thermal peak detection unit, and the modeling analysis of the single heat source equation of the thermal peak detection unit. The obtained simulation and hardware implementation results, compared to a temperature prediction by the GDS method, are given in in Section 3. Section 4 concludes the paper by summarizing our main contributions and findings.

## 2. Theory of Operation of the Proposed on-Chip Thermal Peak Detection Unit

**Figure 1:**
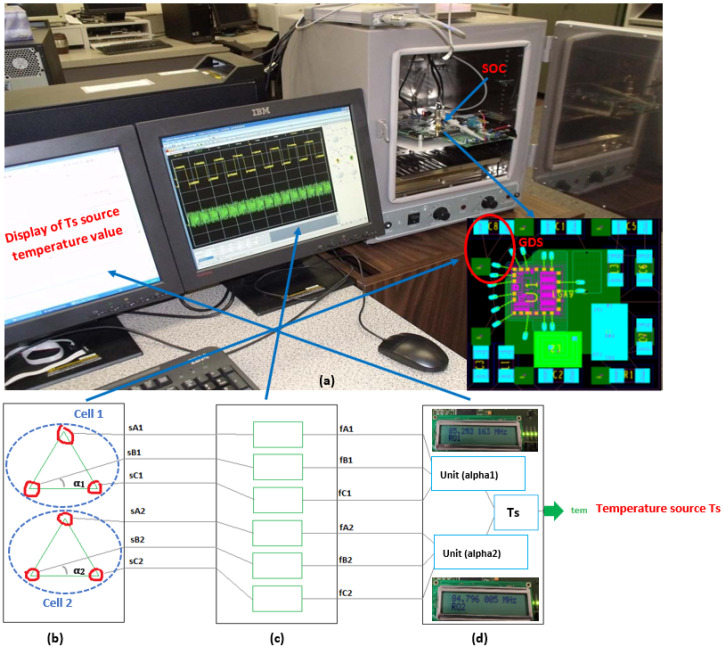
Proposed architecture of a real-time thermal monitoring system based on the GDS method: (**a**) operation of the on-chip thermal peak detection units, (**b**) RO units on an SoC; (**c**) the frequency counters, and (**d**) the computer to analyze the received data.

The heat generated by the operation of various circuits in an SoC creates heat sources at different points on the chip. Numerous point sources can be approximated as a single source (the point at that maximum temperature). Figure 1 illustrates the proposed architecture of a real-time thermal monitoring system based on the GDS method.

Figure 1a shows the three RO units for two cells on the SoC. Figure 1b shows the frequency counters connected to the RO units, and Figure 1c shows the computer used to analyze the received data that completes the thermal peak detection unit. In this system, six well placed thermal sensors transform temperatures into frequency signals. The obtained RO frequency values give information about the heat source $T_{s}$. The temperature measurements are derived from signals sA1, sB1, and sC1 representing sensors A, B, and C, respectively located in cell 1 and from sA2, sB2, sC2 representing sensors A, B, and C, respectively located in cell 2. In Figure 1b, fA1, fB1, and fC1 represent frequencies obtained for A, B, and C, respectively located in cell 1 and fA2; fB2 and fC2 represent frequencies obtained for A, B, and C, respectively located in cell 2. In Figure 1c, the two angles $\tan(\alpha_{1})$ and $\tan(\alpha_{2})$ represent the deviation of the respective triangles of ROs from the heat source $T_{s}$. The two angles $\alpha_{1}$ and $\alpha_{2}$ will be evaluated subsequently based on Equations (1) and (2), in order to evaluate the performance of our method.

### 2.1. The Frequency Counter

**Figure 2:**
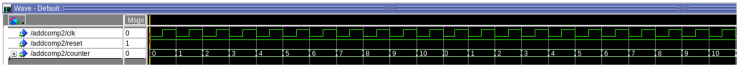
The counter counts until the reset signal is enabled.

As shown in Figure 1b, counters are employed to calculate the oscillation frequency of each RO. As shown in Figure 2, the counter counts until the reset signal is activated and restarts the counter. In the same figure, it counts up to 10 and then the reset signal goes to 1, which restarts the counter at 0.

Our frequency counter based on regression incurs almost no expense. This regression causes almost no overcharge. At run-time, the counter only has to calculate the temperature from a simple formula $T_{s}$ (Equation (3)). This does not affect our performance unlike performance counters that required expensive online computations [13,14,15].

### 2.2. Calculation of the Gradient Angle Units for the GDS Method

#### 2.2.1. The Unit to Compute the Angle α

**Figure 3:**
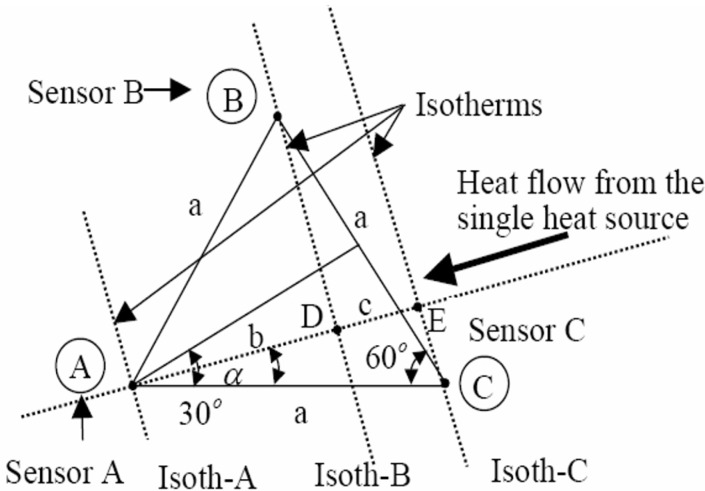
Placement of three sensors cell with α ϵ (0${}^{{}^{\circ}}$, 60${}^{{}^{\circ}}$).

As we already described in Figure 1c, two units are used to calculate the angles $\alpha_{1}$ and $\alpha_{2}$, respectively. Figure 3 illustrates the operating principle of the GDS method. This unit is used to evaluate the position and value of a single heat source on the surface of an SoC. It estimates the geometrical coordinates and temperature of the heat source. To obtain information on the parameters of a single heat source, we provide information on where the thermal peak comes from and the speed at which it evolves. To calculate the temperature of the heat source, a thermal peak detection unit was used. This unit receives the angles $\alpha_{1}$ and $\alpha_{2}$ (computed by the angle calculation unit), the distance h between cell 1 and cell 2, and the oscillation frequency of each RO. The factors requiring special attention during the development of this unit are the number of sensors, the spatial distribution of the heat source, and the network interconnections. This unit provides the angle α that is shown in Figure 3 by according the VHDL code developed in Section 3.

**Figure 4:**
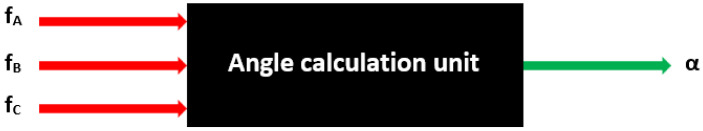
Angle calculation unit.

The GDS method is based on the three embedded sensors adopted to form the first cell. Each cell is composed of three ROs that form a triangle. This unit was designed and tested using VHDL. Equations (1) and (2) define the main functionality of the angle calculation unit that have been simulated and implemented:

(1) $$\tan\left(\alpha_{1}\right)=\frac{2(f_{B1}-f_{A1})}{\sqrt{3}\left(f_{C1}-f_{A1}\right)}-\frac{1}{\sqrt{3}}$$

and

(2) $$\tan\left(\alpha_{2}\right)=\frac{2(f_{B2}-f_{A2})}{\sqrt{3}\left(f_{C2}-f_{A2}\right)}-\frac{1}{\sqrt{3}}$$

where $\alpha_{1}$ and $\alpha_{2}$ indicate the position relative to the heat source. Both formulas were coded in VHDL. As depicted in Figure 4, the angle calculation unit receives the generated frequencies $f_{A}$, $f_{B}$, and $f_{C}$ from the three sensors A, B, and C, respectively, and calculates the tangents and angles (in radians) corresponding to these values.

#### 2.2.2. Source Temperature ($T_{s}$) Calculation

**Figure 5:**
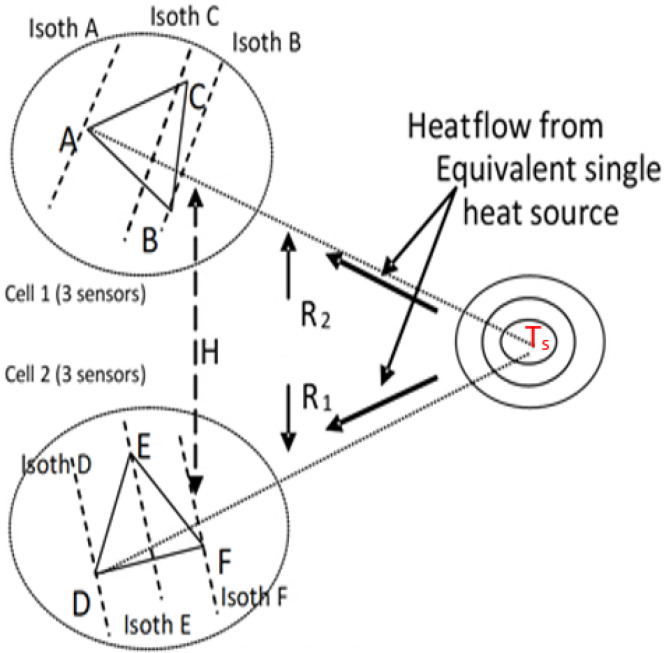
The description and distribution of the sensors’ cells.

In order to obtain the temperature value of a single punctual heat source, we have to calculate the distance between the sensor and this source. Two sensor cells are required for this purpose as depicted in Figure 5. The cells are placed in a given distance (H), and each gives information about angle α ($\alpha_{1}$ and $\alpha_{2}$) in the direction of the heat source. Under the consideration that α is relatively small, we can assume that the heat source and the center of the cells form a triangle in which the length of one side and values of the angles adjacent to this side are known. This means that we can calculate the distances R1 and R2 between the heat source and the sensors. Now we can calculate the temperature gradient along the known distance. By adding distance to the temperature of the sensor, we obtain the temperature of the heat source. Two sensor cells A1, B1, C1 and A2, B2, and C2 are placed in two corners of a monitored layout at the distance H. Hence, the temperature of the heat source can be obtained by Equation (3). Figure 5 shows the description and distribution of the sensors cells:

(3) $$\frac{\frac{H}{a}\left(f_{c1}-f_{A1}\right)\left(\sqrt{3}+\tan\alpha_{2}\right)\left(1+\tan\alpha_{1}^{2}\right)}{\sqrt{3}\left(1-\tan\alpha_{1}\tan\alpha_{2}\right)-\left(\tan\alpha_{1}+\tan\alpha_{2}\right)}+f_{A1}\to T_{s}$$

The effects on performance caused by temperature fluctuations are most often treated as linear scaling, but some sub-micron silicon processes require nonlinear calculations. For this reason, the oscillator network must be activated for a short period of time in order to avoid thermal variations involved by the oscillator itself (self heating).

The temperature accuracy must be ensured with respect to a variation within ±0.2 °C. The source temperature $T_{s}$ is obtained at the output after the unit has processed the input data.The angle calculation unit was implemented and simulated in the ModelSim tool, where different values of $f_{A}$, $f_{B}$, and $f_{C}$ are applied. The results obtained are compared with the calculated ones from the Excel tool. To allow obtaining a synthesizable VHDL code, the tan(α) is calculated not as a real, but as an integer. A factor of 1000 is then used to obtain an integer result that equals 1000 times tan(α). For simulation based validation, we used eight different parameter sets (combinations of $f_{A}$, $f_{B}$ and $f_{C}$).

**Figure 6:**
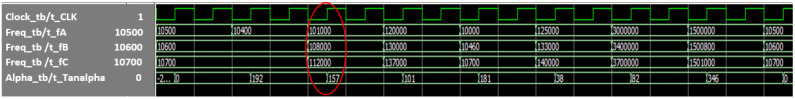
Simulation results of the angle calculation unit.

The results obtained through ModelSim are presented in Figure 6. Using the frequencies received from the frequency counter unit shown in Figure 1b, we can extract the results of the tan(α) from this simulation presented in Figure 6. For example, the frequency collected from sensor A is $f_{A}$ = 101,000 kHz, the frequency collected from sensor B is $f_{B}$ = 108,000 kHz, and the frequency collected from sensor C is $f_{C}$ = 112,000 kHz given a value of tanα = 0.157 (see the part circled in red in Figure 6).

Moreover, the results shown in Figure 6 are compared with the results obtained Excel in Table 1.

As shown in Table 1, the error is small between the Excel and Modelsim models (where line 6 shows the maximum difference of Δ = 0.001 radians), which shows the efficiency of our test bench code developed in VHDL. In addition, we have simulated the temperature calculation unit $T_{s}$ (Equation (3)) using ModelSim. $T_{s}$ is computed as an integer. A factor of 100 is then used to obtain an integer result that equals 10,000 multiplied by $T_{s}$.

**Figure 7:**
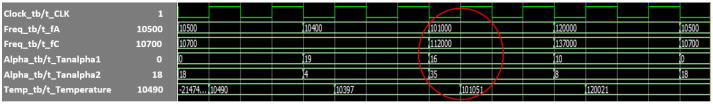
Simulation results of the temperature calculation unit.

The results obtained by ModelSim are presented in Figure 7. Using the received frequencies and the two units that are presented in Figure 1c, we can extract the results of the source temperature $T_{s}$ from this simulation presented in Figure 7; for example, for the $\tan(\alpha_{1})$ = 0.16 and $\tan(\alpha_{2})$ = 0.35, give a value of source temperature $T_{s}$= 10.1051 °C (see the part circled in red in Figure 7).

To validate the results in Figure 7, we compare the results obtained with Excel and the following Table 2, which confirms the validtity of the VHDL model.

A good matching was achieved between the results obtained by Excel and ModelSim. The maximum difference is Δ = 0.06% (as shown in line 2 of Table 2). This small difference is due to the approximations made with the integer model.

## 3. Implementation and Results

**Figure 8:**
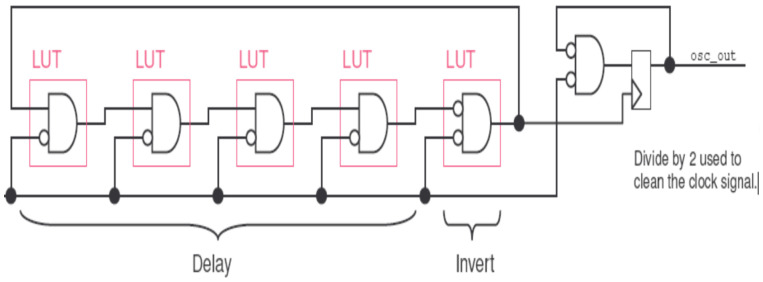
Configuration of the ring oscillator provided by Altera.

For the experiment, we used a DE1 Altera FPGA board and the Quartus Prime software. The FPGA acts as an SoCs with the ability to locate and implement the required ROs for our sensor cells, whereas Quartus Prime provides us with a frequency counter. The ring oscillators are implemented by default in the Altera source code by considering six LookUp Table (LUT) or six delays by default. In addition, Altera’s Quartus Prime configuration defaults to an ambient temperature of 25 °C. This can be seen in Figure 8.

**Figure 9:**
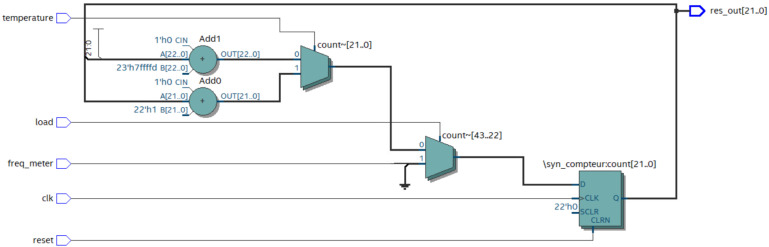
Register Transfer Level structure of the temperature monitoring unit with Quartus Prime.

In this work, we did not focus on the optimization of the delay cell circuits, which is a task left for future work. Based on Figure 8, our experimental design divided into three main parts: simulation, synthesis, and implementation of the VHDL code. Firstly, we developed the VHDL code, and then the Register Transfer Level (RTL) version of the model was generated as shown in Figure 9.

**Figure 10:**
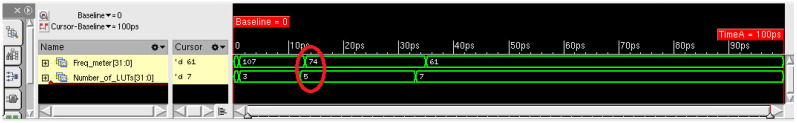
Simulation of the temperature monitoring unit with NClaunch tool.

The logical simulation of the developed VHDL codes is shown in Figure 10, where three correct values of the frequency as a function of the LUT number were obtained (see the part circled in red in Figure 10).

Among the advantages of VHDL coding is the use of the test bench, which allows for verifying the capability of our algorithm to operate the GDS method according to the initial specifications. Test vectors were subsequently created to ensure specific coverage by optimizing the test time, or to minimize performance degradation.

We report that, in this work, we have a conversion time that is around 641 μs, and a conversion rate of 7.4 kHz.

### 3.1. Implementation on the FPGA Board

**Figure 11:**
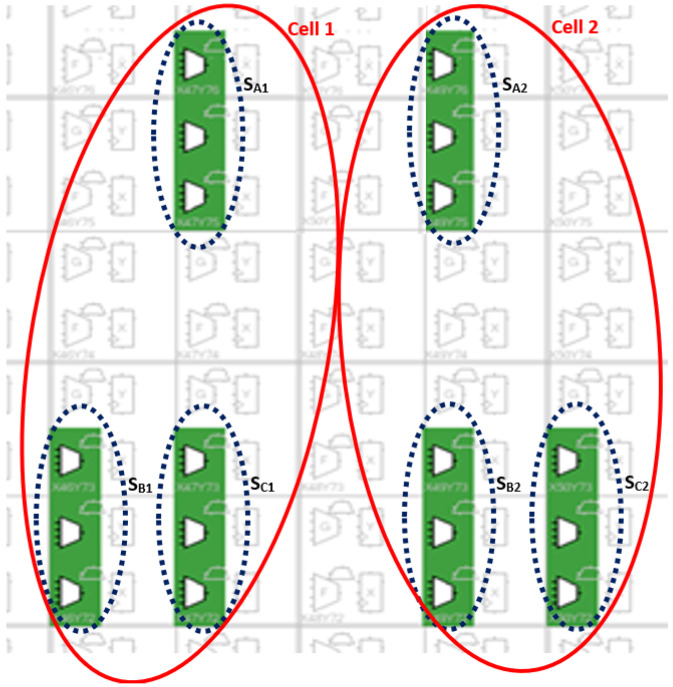
Physical location of the six ROs sensors on the FPGA.

It is worth noting that the Quartus Prime software allows a manual placement of the circuit on the FPGA. Figure 11 shows the physical location of the six ROs (The RO occupies a 0.39 μm${}^{2}$) on the FPGA.

**Figure 12:**
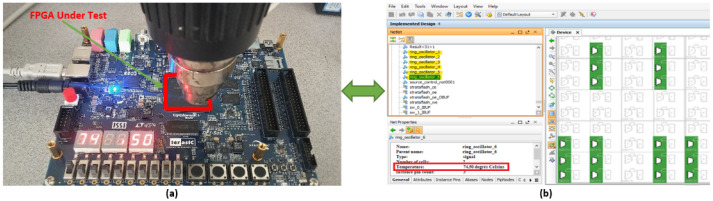
Read temperature by: (**a**) the FPGA board and (**b**) the Quartus Prime tool.

Figure 11 represents the implementation of the six ROs on the FPGA board. After downloading the VHDL code, the program was running and the outputs were displayed in Figure 12.

The clock is set to 50 MHz, and a dryer is used to increase the temperature of the DE1 (Development and Education) FPGA board. Figure 12 shows the obtained temperature results.

Figure 12a shows the temperature displayed by FPGA under test. Figure 12b shows all the information pertinence’s on the six RO sensors by the Quartus Prime tool. Our experimental results show that the detected temperature from the six ROs is 74.50 °C.

As mentioned in the Introduction, the purpose is to detect thermal peaks using the GDS method, and we tried to vary the temperature to see its influence on the frequency. The obtained values are reported in the Table 3 below:

Table 3 shows that, as the temperature increases, the frequency decreases by a constant value of 2.07 MHz for each 10 °C difference.

In order to validate the results found in Figure 12 and show the ability of the proposed GDS method to monitor the temperature in real-time, we then proceeded with thermal camera measurements as reported in the next section.

### 3.2. Experimental Measurements by the Thermal Camera

In this work, a JENOPTIK infrared camera [16] is used to capture the board’s temperatures. It offers a spectral range of 7.5 to 13 μm with a frequency of 61 MHz and an image resolution of 320 × 240 pixels. It also offers an accuracy of ±0.2 °C.

The software used for this camera is the IRT analyzer. It is installed on a computer on which we make the acquisition and extraction of thermal measurements.

**Figure 13:**
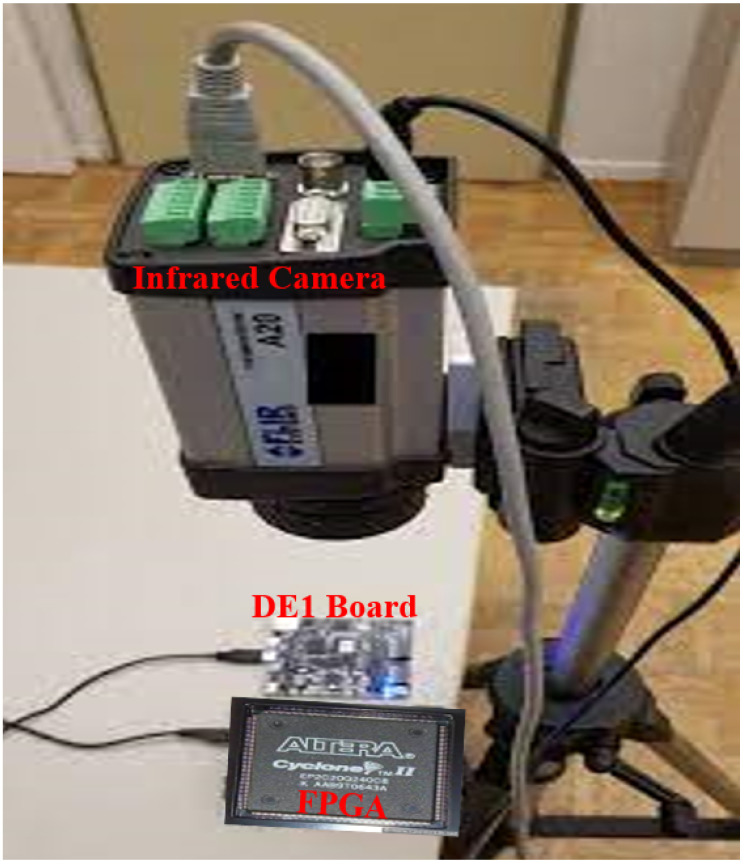
Thermal measurement system exploiting an infrared camera.

Figure 13 shows the whole setup used for measurement by infrared thermography.

To extract the temperature values from the thermal camera, we used an image processing tool (IRT analyzer) that allows us to analyze the raw data collected by the infrared camera and convert them into digital data that can be used in the validation process.

**Figure 14:**
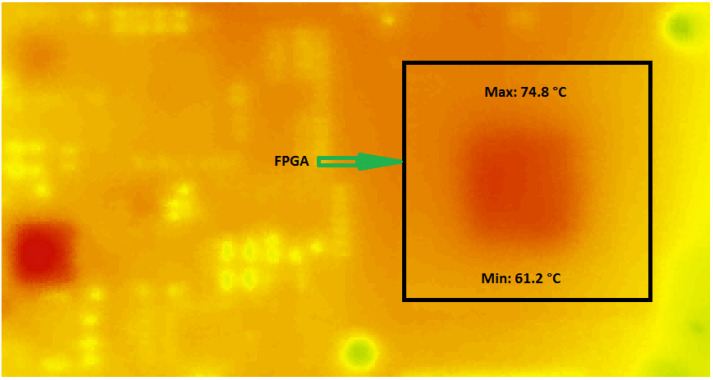
Temperature measurement of the FPGA board by thermal camera.

Figure 14 shows an overview of the IRT analyzer tools. The use of the high precision IRT tool greatly increases the sensitivity and quality of the data received by the thermal camera, as well as the ability to analyze the experimental measurements.

The results displayed in Figure 14 are obtained using the generation of the library integrated under the IRT tool and the measurements that are performed via the thermal camera.

Figure 14 shows the areas where the highest temperature was observed (74.80 °C) on the DE1 map. The realization of a temperature measurement system by infrared thermography allowed us to extract the thermal experimental values with an emissivity equal to 1.

To validate the proposed method, we proceed to a comparison between thermal camera and FPGA measurement where the following Table 4 summarizes the obtained results.

This table clearly shows that the temperature values are almost the same for both results. The maximum error is about 0.3 °C, which indicates a good result for the validation of our method. Table 5 summarizes the GDS method on integrated circuits compared to similar works.

The performance of the proposed method based on ROs is reported in Table 5. Our results are compared to those of other methods’ solutions presented in [17,18,19,20,21] (see Table 5). We can deduce that our work has significant potential, especially in terms of error and real-time data processing, which is not presented anywhere else. This performance includes the speed of obtaining the information that will allow us to intervene in real-time, especially since the majority of high-throughput SoCs so far do not have effective ways to predict thermal peaks and assess temperature in real-time.

## 4. Conclusions

This paper proposes a new thermal monitoring method that exploits in-situ temperature measurements derived from ring oscillator (RO) frequency measurements for thermal peak detection. A thermal peak detection unit was designed. Frequencies are converted to temperatures using the proposed models. In order to verify the presented method, we implemented three RO sensors for each cell on an FPGA board and temperature measurements were validated with a thermal infrared camera. A maximum error of 0.3 °C was observed between measured and validated temperatures.

The objective of this research is to obtain information about thermal peaks in real-time, which helps designers to react timely to possible hazards caused by thermal hot spots. The paper also provides other benefits such as helping to characterize and locate thermal peaks. Through simulations and experiments, it was shown that ring oscillators (ROs) are capable of providing in-situ temperature measurements.

This work offers a solution that allows for identifying thermal induced stress and local overheating of integrated systems that are major concerns for integrated circuits’ designers.

A limitation of our proposal is that the integrated sensors are designed in CMOS technology, which is limited by the maximum operation temperature (around 120 °C). In addition, the sensitivity of the integrated sensors must be improved by improving the circuitry of ring oscillators. Our future work will focus on improving the sensitivity of integrated sensors and optimizing the accuracy of the proposed technique.

## Figures and Tables

**Table 1:** Comparison between results obtained with ModelSim and Excel.

$f_{a}$ (kHz)	$f_{b}$ (kHz)	$f_{c}$ (kHz)	tan(α) with Excel	tan(α) with ModelSim
10,500	10,600	10,700	0	0
10,400	10,600	10,700	0.192	0.192
101,000	108,000	112,000	0.157	0.157
120,000	130,000	137,000	0.101	0.101
10,000	10,460	10,700	0.181	0.181
125,000	133,000	140,000	0.037	0.038
3,000,000	3,400,000	3,700,000	0.081	0.082
1,500,000	1,500,800	1,501,000	0.345	0.346

**Table 2:** Results obtained with the ModelSim and Excel.

$f_{a}$ (kHz)	$f_{c}$ (kHz)	$\tan(\alpha_{1})$	$\tan(\alpha_{2})$	$T_{s}$ with Excel	$T_{s}$ with ModelSim
10500	10700	0	0.18	1.0486	1.0490
10,400	10,700	0.19	0.04	1.0391	1.0397
101,000	112,000	0.16	0.35	10.1046	10.1051
120,000	137,000	0.10	0.08	12.0020	12.0021

**Table 3:** Results of the oscillation frequency versus temperature obtained.

Temperature (°C)	Frequency (MHz)
25	99.88
30	98.21
40	96.12
50	94.18
60	92.08
70	90.20
80	88.10
90	86.04
100	84.17
110	82.12
120	80.02

**Table 4:** Comparison between thermal camera and FPGA measurement results.

Measurements	by Thermal Camera	by GDS	Error Rates (°C)
Temperature (°C)	74.80	74.50	0.3

**Table 5:** Comparison of the performance of the GDS method on integrated circuits compared to similar works.

References	Proposed	[17]	[18]	[19]	[20]	[21]
Method	GDS ${}^{\left(a\right)}$	STS ${}^{\left(b\right)}$	FEM ${}^{\left(c\right)}$	DTM ${}^{\left(d\right)}$	TSERO ${}^{\left(e\right)}$	DTS ${}^{\left(f\right)}$
Temperature (°C)	110 ${}^{\left(*\right)}$	75	80	100	120	125
Error (°C)	0.3	0.8	3.7	2.8	2.9	0.6
Real-time Monitoring	Yes	No	No	No	No	No

${}^{\left(a\right)}$ Gradient Direction Sensor (GDS); ${}^{\left(b\right)}$ Smart Temperature Sensor (STS); ${}^{\left(c\right)}$ Finite Element Method (FEM); ${}^{\left(d\right)}$ Dynamic Thermal Management (DTM); ${}^{\left(e\right)}$ Temperature Sensor Employs two Ring Oscillators (TSERO); ${}^{\left(f\right)}$ Digital Temperature Sensor (DTS). ${}^{\left(*\right)}$ Maximum measured temperature.

## Data Availability

The data that support the findings of this study are available from the corresponding author upon reasonable request.
